# Run-Based Tests Performed on an Indoor and Outdoor Surface Are Comparable in Adolescent Rugby League Players

**DOI:** 10.3390/sports13100351

**Published:** 2025-10-04

**Authors:** Michael A. Carron, Vincent J. Dalbo

**Affiliations:** 1 School of Health, Medical and Applied Sciences, Central Queensland University, Rockhampton, QLD 4702, Australia; m.carron@cqu.edu.au; 2 Health Education Lifestyle and Performance (HELP) Laboratory, St Brendan’s College, Yeppoon, QLD 4703, Australia

**Keywords:** test–retest reliability, youth, performance, strength and conditioning, exercise

## Abstract

At non-professional levels of rugby league, run-based tests are commonly performed on outdoor turfed fields and on indoor multipurpose sport surfaces, and results are monitored to gauge player performance and progression. However, test–retest reliability has not been conducted on indoor surfaces in adolescent rugby league players, and no research has examined if results obtained on outdoor and indoor surfaces are comparable for practitioners. Adolescent, male, rugby league players (N = 15; age = 17.1 ± 0.7 years) completed a 20 m linear sprint test (10- and 20 m splits), 505-Agility Test, and Multistage Fitness Test (MSFT) weekly for three consecutive weeks. Absolute (coefficient of variation (CV)) and relative (intraclass correlation coefficient (ICC)) reliability of each run-based test performed on the indoor surface was quantified. Dependent *t*-tests, Hedges *g*, and 95% confidence intervals were used to examine if differences in performance occurred between indoor and outdoor surfaces. Effect size magnitudes were determined as *Trivial*: <0.20, *Small*: 0.20–0.49, *Medium*: 0.50–0.79, and *Large*: ≥0.80. All tests were considered reliable on the indoor surface (CV < 5.0%; ICCs = *moderate-good*) except for the 505-Agility Test (CV = 4.6–5.1%; ICCs = *poor*). Non-significant (*p* > 0.05), *trivial* differences were revealed between surface types for 10 (*g* = 0.15, 95% CI = −0.41 to 0.70) and 20 m (*g* = 0.06, 95% CI = −0.49 to 0.61) sprint tests, the 505-Agility Test (Right: *g* = −0.53, 95% CI = −1.12 to 0.06; Left: *g* = −0.40, 95% CI = −0.97 to 0.17), and the MSFT (*g* = 0.25, 95% CI = −0.31 to 0.81). The 10 and 20 m linear sprint test and MSFT have acceptable test–retest reliability on an indoor multipurpose sport surface, and practitioners may compare results of run-based tests obtained on an outdoor and indoor surface.

## 1. Introduction

Successful performance in rugby league is highly dependent on several run-based skills such as linear speed, change of direction, and aerobic capacity [1]. As a result, practitioners (i.e., coaches, sports performance, and sports science staff) seek to enhance on-field player performance by creating structured training protocols aimed at improving linear speed, change of direction, and aerobic capacity of players [2]. To ensure that the structured training protocol elicits positive training adaptations, practitioners periodically assess the effects of training on player performance of the variables they are seeking to improve [2,3]. As a result, it is common practice for rugby league practitioners to obtain several measures of linear speed (commonly assessed with 10 and 20 m sprint tests), change of direction (commonly assessed with the 505-Agility Test), and aerobic capacity (commonly assessed with the Multistage Fitness Test (MSFT)) over the duration of the season to monitor the efficacy of their training protocol [4,5].

In rugby league, particularly at the high school level, linear speed, change of direction, and aerobic capacity testing primarily occurs on outdoor turfed fields. In this regard, numerous studies have reported 10 m linear sprint speed, 20 m linear sprint speed, 505-Agility Test, and MSFT performance to be reliable on outdoor turfed fields in adolescent, male, rugby league players [6,7,8]. However, conducting run-based tests on outdoor turfed surfaces introduces unnecessary error into test results via extraneous variables such as weather, varied surface conditions such as turf type (e.g., various types of grass and variability in turf height), and variations in surface stiffness (e.g., moisture affecting stiffness) of the turfed surface [9,10]. Furthermore, given that the Australian adolescent rugby league season (also known as schoolboy rugby league) spans from summer through winter (i.e., the match schedule occurs from May to September), extraneous variables influencing outdoor turfed surface conditions are inevitable. Specifically, practitioners are likely to make assessments on player adaptations from outdoor test results that were collected in conditions that had substantial variations in heat [11], solar exposure [12], and moisture [13] due to rain and/or dew which effect traction, friction, and surface stiffness [14]. Each condition can affect the physiological (i.e., core body temperature and heart rate) [15] and biomechanical (i.e., ground reaction force and friction) performance of the player [9,10], which can influence test results [9,13]. Despite the limitations of conducting run-based tests on outdoor turfed surfaces, outdoor testing is the preferred mode of testing of adolescent rugby league practitioners and can be justified by the logic that rugby league is played on outdoor turfed surfaces.

Even though it is the preference of adolescent rugby league practitioners to conduct run-based tests on outdoor turfed surfaces, it is not uncommon for testing to occur on indoor multipurpose sport surfaces primarily due to weather events such as rain, snow, or lightning. As a result, it is common for adolescent rugby league practitioners to gauge player performance and progression with results of run-based tests obtained on outdoor turfed surfaces and indoor multipurpose sport surfaces. This practice raises two questions. First, are the results of run-based tests obtained on an indoor multipurpose sport surface reliable in adolescent rugby league players as no found research has been published on this topic. Second, assuming the results of run-based tests performed on an indoor multipurpose sport surface are reliable in adolescent rugby league players, can practitioners meaningfully compare results of run-based tests obtained on an outdoor turfed surface and an indoor multipurpose sport surface which is commonly occurring in practice.

## 2. Materials and Methods

### 2.1. Participants

The first aim of our study was to quantify the test–retest reliability of the 10 m sprint test, 20 m sprint test, 505-Agility test, and MSFT on an indoor multipurpose sports surface in our sample. As a result, no power analysis was required for this study aim. The second aim of our study was to determine if results from a 10 m sprint test, 20 m sprint test, 505-Agility test, and MSFT performed on an outdoor turfed field and an indoor multipurpose sports surface could be meaningfully compared by practitioners to make informed decisions about player performance and progression. As a result, we conducted an *a priori* power analysis using G*Power (3.1.9.7; Heinrich Heine University, Dusseldorf, Germany). Based on the statistical test, differences between two dependent means (matched pairs), a two-tailed test, an effect size of 0.80, an α error probability of 0.05, and a power (1-β error probability) of 0.80, the required sample size was calculated as 15. Therefore, our study was adequately powered to detect if a *large* difference occurred in the performance time of each run-based test performed on the two surface types.

Adolescent, male, rugby league players (N = 15, age: 17.1 ± 0.7 years; height: 179.96 ± 5.95 cm; mass: 83.36 ± 14.59 kg) from the same team volunteered to partake in this study. Players were recruited to participate if they were selected to compete in the first-grade schoolboy competition (e.g., equivalent to the varsity team in American high schools) and were free from injury and illness. All players had a minimum of six months of a consistent training history (i.e., rugby league skills and development training and technical/safety introductions to resistance training) and were familiarized with all tests to be completed at least once prior to the first testing session. Written informed assent was obtained from all participants and written informed consent was obtained from a legal guardian of each participant prior to the commencement of this study. The study protocol was approved by the institution’s Human Research Ethics Review Committee prior to the commencement of the study (#0000023570).

### 2.2. Study Design

**Figure 1:**
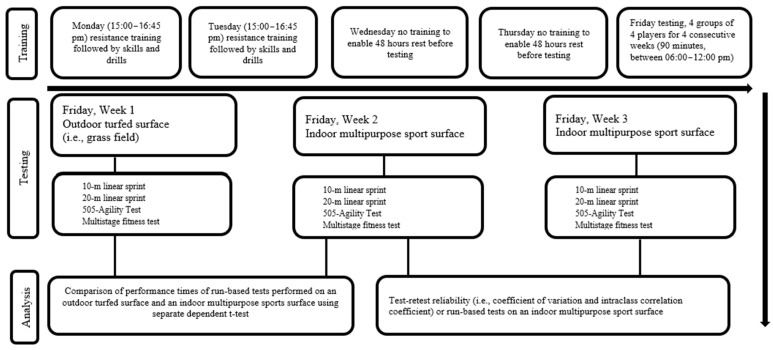
Schematic of the study design.

A within-participant, repeated measure, crossover design was employed for this study, which occurred at the end of the in-season training phase of the season. Players were partaking in three gym sessions (resistance training occurred on Monday, Wednesday, and Friday) and two field sessions (skills and drills training occurred on Monday and Wednesday) per week. Training occurred as follows: Monday (resistance training 15:00–15:50 followed by a field session 15:50–16:45), Wednesday (resistance training 15:00–15:50 followed by a field session 15:50–16:45), and Friday (06:00–07:00, resistance training). Data collection for this study over three weeks. During the three-week data collection period, participants trained as specified on Monday but did not train on the Wednesday (to allow for 48 h rest), and the Friday training session was replaced by testing (06:00–12:00). This modification to the training schedule allowed for a 48 h rest prior to each training session and testing sessions (Figure 1). Testing occurred in the following order: Week 1: Run-based tests performed on an outdoor turfed surface (i.e., a grass field). Weeks 2 and 3: Run-based tests performed on an indoor multipurpose sports surface. The results of run-based tests conducted during weeks 2 and 3 allowed for the examination of test–retest reliability of each run-based test on an indoor multipurpose sports surface (Aura Sports, Polyurethane Flooring; Ipswich, QLD, Australia). The results of the run-based tests conducted during weeks 1 and 2 allowed for the comparison of tests performed on an outdoor turfed surface and an indoor multipurpose sports surface. It is common practice for the reliability of run-based tests to be evaluated on different days within a timeframe of seven days to limit or prevent results of subsequent tests from being influenced by maturation in younger cohorts or a training effect in those undertaking structured training [16].

Each Friday of the testing period, players reported to the testing facility and performed a consistent 15 min warm-up protocol that was delivered prior to each testing session and was completed by all players. The warm-up comprised five minutes of light aerobic cycling, several mobility exercises which targeted major functional movements, and dynamic stretches of the lower body for four minutes. Thereafter, movement preparation exercises with body weight or resistance bands (i.e., squat, lunge, Pallof press, and single-arm row) were performed for four minutes, and a football was repeatedly passed over 10 m intervals while moving at different intensities for two minutes. Tests were always administered in the following order: 20 m linear sprint tests (from which the 10 m sprint time split was derived), 505-Agility Test, and the MSFT. The order of testing follows the recommendations of the National Strength and Conditioning Association (NSCA) [17].

### 2.3. Methods

Players were instructed to consume their typical diet, refrain from vigorous activity, and continue with their normal daily schedules (i.e., nutrition, hydration, sleep, and school participation) for 48 h prior to the commencement of each testing session [6,18]. Testing sessions were conducted in three groups of four players and one group of three players. Each group of players completed testing in 90 min sessions as follows: group 1: 06:00–07:30; group 2: 07:30–09:00; group 3: 09:00–10:30; and group 4: 10:30–12:00. Each group of players was assigned to the same scheduled time for each testing session to reduce the effects of circadian rhythms [19], hormonal fluctuations [20], and feeding [21] on performance. For each testing session, participants wore regular rugby league attire (t-shirt and shorts), but footwear was altered to suit the change in surface conditions. During outdoor testing, participants wore cleats; during indoor testing, participants wore running shoes. Participants were instructed to wear the same pair of running shoes during each indoor testing session.

### 2.4. Linear Speed

Linear speed was assessed with 10 m and 20 m linear sprint times (s). Players were positioned with the toes of their leading foot placed 50 cm behind the first timing gate on marking tape in a split stance before initiating each sprint [22]. Each player completed three 20 m sprints with each 20 m sprint commencing upon their own volition. Single-beam electronic timing gates (SmartSpeed Pro, Fusion Sport; Brisbane, QLD, Australia) were used to record sprint times. Single-beam electric timing gates were set at 0 m, 10 m, and 20 m (at a height of 1 m) to obtain a measure of 10 m and 20 m sprint times (s). Performance times (s) were retrieved immediately after each attempt via the SmartSpeed application (version 1.0.4) for 10 m and 20 m splits to the nearest 0.001 s. Between each attempt, 60 s of passive standing rest was allocated to separate attempts, with the fastest of the three attempts for each split (10 m and 20 m) recorded for analysis [6].

### 2.5. Change-of-Direction

Change-of-direction was assessed with the 505-Agility test. Players began each attempt standing with the toes of their lead foot on marking tape set at the 0 m point. Players were expected to move as fast as possible on their own volition in a forward direction for 15 m before turning 180 degrees back towards the starting point, and accelerating for a further 5 m [1]. One single-beam electronic timing gate (SmartSpeed Pro, Fusion Sport; Brisbane, QLD, Australia) was positioned 10 m from 0 m starting point to record performance time to the nearest 0.001 s. Timing commenced as participants passed through the timing gate covering an additional 5 m before turning and passing the timing gate to complete the test. Performance was assessed separately for the left and right leg to initiate the COD (plant leg), requiring players to complete six total attempts in the same order on each occasion (performed first, as a right lead leg for three attempts and then left lead leg for the final three attempts, with the lead leg being the foot to make the turn). The fastest of the three attempts for each leg was recorded for analysis.

### 2.6. Aerobic Capacity

Aerobic capacity was assessed with the MSFT to estimate maximal oxygen uptake (VO_2max_ [mL·kg^−1^·min^−1^]), following an established procedure and equation [23]. The MSFT requires participants to perform repeated 20 m shuttle runs with progressive increases in speed dictated by audio cues. The MSFT concluded when players voluntarily stopped or failed to complete the necessary distance as signaled by audio cues across two successive shuttles. During testing on the outdoor turfed surface, players completed the MSFT on a regulation, grass, rugby league field with clearly marked lines to indicate the 20 m distance. During indoor testing, players completed the MSTF on an indoor multipurpose sports surface with clearly marked lines indicating the 20 m distance.

### 2.7. Reliability and Levels of Acceptability

Reliability of tests is often assessed by quantifying the coefficient of variation (CV) and intraclass correlation coefficient (ICC) [24]. CV represents the absolute reliability of a test and quantifies the random error between repeated measurements. CV is most relevant when assessing changes in the outcome of a test over time (i.e., the repeatability of the result in regard to average performance) [16]. In contrast, ICC represents relative reliability, which quantifies the amount of variability in the measurement that was due to differences between participants (i.e., the repeatability of the test in regard to how participants finish in the same order/hierarchy when a test is repeated over time) [16]. Using established criteria, a CV of <5.0% is considered reliable for a 10 m sprint, 20 m sprint, and 505-Agility Test, while a CV of <10.0% is considered reliable for the MSFT [25]. Given no specific thresholds of ICCs for run tests exist, ICCs were evaluated using generalized established criteria of *poor*: ≤0.50, *moderate*: 0.50 to 0.74, *good*: 0.75 to 0.90, or *excellent*: ≥0.90 and were interpreted based on the ICC value without considering the 95% confidence interval (CI) [26]. The CV and ICC of run-based tests performed on an indoor multipurpose sport surface were determined using the best attempt of each trial that occurred during each testing session (e.g., the best 10 m sprint time obtained from testing during week 2 and the best 10 m sprint time obtained from testing during week 3).

### 2.8. Statistical Analyses

To accomplish the first aim of our study, we quantified the CV and ICC with a 95% CI [26] for each run-based test performed on an indoor multipurpose sport surface. The CV of each run-based test was quantified using an established formula [27] with calculations being performed in Microsoft Excel (version 15, Microsoft Corp; Redmond, WA), while the ICC and 95% CI were quantified using IBM SPSS software (version 24 IBM Corp; Armo, NY, USA). To accomplish the second aim of our study, we compared the results of each run-based test performed on an outdoor turfed surface and an indoor multipurpose sport surface using separate dependent *t*-tests (i.e., outdoor turfed surface test results obtained during week 1 were compared to indoor multipurpose sport surface test results obtained during week 2). Prior to making any comparisons, each dependent variable was tested for normality with separate Shapiro–Wilk tests. Each dependent variable was normally distributed (*p* > 0.05) except for the 10 m sprint test performed on the outdoor turfed surface (*p* < 0.05). As a result, we visually inspected the distribution of data for 10 m sprint performance using Q–Q plots and determined the data to be reasonably normally distributed. As a result, all data were reported as means and standard deviations (SD). Significance testing (i.e., *p*-values) was utilized to determine if a repeatable difference was present for each run-based test performed on an outdoor turfed surface or indoor multipurpose sport surface with significance set *a priori* at *p* < 0.05. An effect size and 95% CI were utilized to determine if a meaningful difference was present for each run-based test performed on an outdoor turfed surface or an indoor multipurpose sport surface. Due to the sample size, Hedges’ *g* was selected as the effect size measure [28] and was reported with a 95% CI. The effect size magnitude of Hedges’ *g* was determined as *Trivial*: <0.20, *Small*: 0.20 to 0.49, *Medium*: 0.50 to 0.79, or *Large*: ≥0.80; however, if the 95% CI overlapped 0, the effect size magnitude was defined as *trivial*. Dependent *t*-tests were conducted with Jamovi (version 2.3.28; the Jamovi project) while Hedges’ *g* and the 95% confidence intervals were quantified in Microsoft Excel (version 15, Microsoft Corp; Redmond, WA, USA) as follows:

Step 1: Quantification of Cohen’s d [28]

Cohen’s *d* = (Time Point A Mean − Time Point B Mean)/Pooled Standard Deviation

We then calculated (Outdoor testing time point − initial (week 2) indoor time point)/Pooled Standard Deviation

Step 2: Quantification of Hedges’ g [28]

Hedges *g* = d*(1 − (3/(4*(Number of Pairs − 1) − 1)))

where d = Cohen’s d

Step 3: Quantification of the 95% Confidence Interval for Hedges’ g

95% CI for Hedges’ *g* = g ± (t_α/2_*SE_g_)

where *g* = The effect size of Hedges’ g

t_α/2_ = The critical value from a dependent samples t-distribution table (*N* = 15 t_2.131_ [29]

and df = N − 1)

SE_g_ = Standard error of Hedges’ *g* quantified as follows:

SE_g_ = √((1/Number of Pairs) + (g^2/(2*Number of Pairs))))

## 3. Results

### 3.1. Test–Retest Reliability

Table 1 displays reliability statistics for each run-based test. All tests performed on an indoor multipurpose sport surface displayed an acceptable CV other than the 505-Agility test with a left leg lead (CV = 5.1%). The ICCs on an indoor multipurpose sport surface ranged from *poor* (505-Agility test, right and left leg) to *good* (10 and 20 m sprint).

### 3.2. Assessment of Significant and/or Meaningful Differences in Run-Based Tests Occurring on an Outdoor Turfed Field and an Indoor Multipurpose Sport Surface

Non-significant, *trivial* differences were revealed for each run-based test performed on an outdoor turfed surface and an indoor multipurpose sport surface (Table 2).

## 4. Discussion

Commonly utilized run-based tests in adolescent rugby league include the 10 m sprint test, 20 m sprint test, 505-Agility Test with a left and right lead leg, and the MSFT [6]. Each of these run-based tests has previously been deemed reliable on an outdoor surface [6,8] but no research has examined the reliability of these tests in adolescent rugby league players on an indoor surface. Moreover, no study has examined whether practitioners can make meaningful comparisons between run-based tests performed on outdoor and indoor surfaces despite commonly occurring in practice. Findings from our study suggest that the 10 m sprint, 20 m sprint, and MSFT can be reliably performed on an indoor multipurpose sport surface, as each of these tests yielded an acceptable CV (CV <5.0%) and *moderate* to *good* ICC values (ICC range: 0.63 to 0.86). Results from our data suggest the 505-Agility Test (right and left lead leg) should be used with caution on an indoor multipurpose sport surface due to poor absolute reliability (CV right lead leg: 4.6%, CV left lead leg: 5.1%) and *poor* relative reliability (ICC right lead leg: 0.19, ICC left lead leg: 0.21). Results from the dependent *t*-tests suggest practitioners can make comparisons between data collected on an outdoor turfed surface and an indoor multipurpose sport surface for the 10 m sprint, 20 m sprint, 505-Agility Test, and MSFT due to the lack of significant (*p* ≥ 0.05) or meaningful differences (i.e., the effect size magnitude was *trivial* for each comparison). However, we do not recommend performing the 505-Agility Tests on an indoor multipurpose sport surface due to displaying poor test–retest reliability.

Test–retest reliability of linear speed tests in adolescent, male rugby league players has previously been established on outdoor turfed surfaces [6,7]. Specifically, Carron et al. [6] assessed the test–retest reliability of the 10 m (CV = 2.1%, ICC = 0.704) and 20 m linear sprint (CV = 1.4%, ICC = 0.869) tests in adolescent, male, rugby league players (N = 50; 16.2 ± 1.3 years), while Dobbin et al. [8] assessed the test–retest reliability of 10 m (CV = 4.2%, ICC = 0.81) and 20 m linear sprint (CV = 3.6%, ICC = 0.78) tests in academy-level, male, rugby league players (N = 50, 17.1 ± 1.1 years). Moreover, Darrall-Jones et al. [7] assessed the CV of 10 m (CV = 3.1%) and 20 m linear sprint tests (CV = 1.8%) in a sample of adolescent, male rugby players (N = 28, 17.7 ± 0.6 years), but their results reflect data collected in academy rugby league (n = 14) and rugby union (n = 14) players reported together (N = 28).

Results from our study revealed the 10 m sprint (CV = 1.9%, ICC = 0.76) and 20 m sprint (CV = 1.4%, ICC = 0.86) to be reliable on an indoor multipurpose sport surface and are comparable to previously published results obtained on an outdoor surface in adolescent, male, rugby league players [6]. However, results from our study suggest the 505-Agility Test performed on an indoor surface to be unreliable, as CVs ranged from 4.6% (right foot lead) to 5.1% (left foot lead). Given that a CV of <5.0% is considered acceptable [28] and we had a small sample size (N = 15), future research conducted with a larger sample size may reveal the 505-Agility Test to have an acceptable CV on an indoor surface. However, we found the 505-Agility Test to present *poor* ICCs with a right foot lead (ICC = 0.19, 95% CI = −0.34 to 0.63) and left foot lead (ICC = 0.21, 95% CI = −0.32 to 0.64). To our surprise, our findings suggest when assessing tests of change of direction, specifically with the 505-Agility test, it may be more suitable to use an outdoor turfed surface, as previous investigations conducted in adolescent, male, high school rugby league players found the 505-Agility test to be reliable (right foot lead: CV = 1.85%, ICC = 0.787; left foot lead: CV = 2.07%, ICC = 0.860) when obtained on an outdoor surface [6]. We speculate that the degree of surface stiffness likely affects the consistency of change of direction movements when performed on an indoor multipurpose sport surface. Specifically, friction and traction are integral for cutting maneuvers [9] and the malleability (i.e., softness) of the turfed surface likely enables players to plant and stabilize their footing more efficiently by way of surface manipulation [30], particularly when wearing cleats. In this way, players may alter the surface and utilize ground reaction forces to articulate their mechanical power effectively when performing the 180-degree turn expressed in the 505-Agility test [31]. Moreover, stiffer surfaces yield less force loss via deformation of the surface resulting in improved proprioceptive feedback assisting mechanical and technical efficiency [32] when performing the sprinting component of the 505-Agility test [31]. Conversely, non-malleable surface types, such as an indoor multipurpose sport surface may introduce a slip factor negatively influencing the reliability of test results.

We found the MSFT to be reliable when performed on an indoor surface in adolescent, male, high school rugby league players (CV = 4.1%, ICC = 0.63, 95% CI = 0.19 to 0.86, *Moderate*). Our results differ from a previous study that investigated the reliability of the MSFT in male, adolescent, high school rugby league players on an outdoor turfed surface (CV = 3.04, ICC = 0.84, *Good*) [6]. The negligible differences between the reliability (CV and ICC) of the MSFT we found on an indoor multipurpose sport surface, and that which Carron et al. [6] reported on an outdoor turfed surface, suggest that practitioners can make meaningful comparisons of MSFT results obtained on these surfaces. However, due to the extraneous variables introduced by outdoor testing, best practice would suggest assessing MSFT performance on an indoor multipurpose sport surface when possible.

If accuracy is the primary concern of practitioners, the 10 m sprint, 20 m sprint, and MSFT should be performed on an indoor multipurpose sport surface in adolescent, male, rugby league players. Conducting these run-based tests on an indoor multipurpose sport surface would aid in standardizing run-based tests, resulting in greater homogeneity of testing procedures across studies and allowing for more accurate comparisons between studies. Moreover, the adoption of standardized testing procedures would enable practitioners to more accurately compare results from test outcomes obtained in practice to test outcomes presented in research. Due to the 505-Agility Test having poor reliability in our study, we cannot currently recommend performing the 505-Agility Test on an indoor multipurpose sport surface. However, future studies should attempt to replicate our findings, as we had a small sample size and we appear to be the first to report on the reliability of run-based tests on an indoor multipurpose sport surface in adolescent, male, rugby league players. Therefore, more research should be conducted before definitive conclusions about the reliability of run-based tests in adolescent, male, rugby league players and other populations can be confirmed.

Notably, we recommended that practitioners, who are using run-based tests to monitor player progression over time, perform run-based tests on an indoor multipurpose sport surface, when possible, as fewer extraneous variables are introduced to the testing protocol compared to testing on an outdoor turfed surface. In this vein, the assessment of test–retest reliability of run-based tests has previously occurred within a seven-day period [6,8]. As a result, test–retest reliability outcomes of run-based tests performed on an outdoor turfed surface are likely to occur under similar external conditions such as temperature, humidity, solar exposure, and surface conditions. However, if monitoring performance of run-based tests take place over the duration of a year, in most climates, factors such as temperature, humidity, solar exposure, and surface conditions will vary greatly from summer to winter. Our study was conducted in a subtropical climate and the average 9 a.m. conditions in February (Season: temperature: 27.2 °C, humidity: 75%, dew point: 22.1 °C, wind speed: 17.0 km/h, solar exposure: 21–24 kWh/m^2^) [33,34] were notably different to the average 9 a.m. conditions in September (Season: Sprint, temperature: 21.9 °C, humidity: 69%, dew point: 15.7 °C, wind speed: 13.7 km/h, solar exposure: 21–24 kWh/m^2^) [33,34].

We conducted this study as it is common for practitioners to gauge player performance and progression utilizing run-based test results obtained on outdoor turfed surfaces and indoor multipurpose sport surfaces. Therefore, we determined if there were significant or meaningful differences in performance times for the 10 m sprint test, 20 m sprint test, 505-Agility Test, and MSFT. We found there to be no significant (*p* ≥ 0.05) or meaningful (a *trivial* effect size magnitude was found for each test) differences between performance outcomes of each run-based test performed on an outdoor turfed field or an indoor multipurpose sport surface. To our surprise, our results suggest that, if required, practitioners may compare results of run-based tests obtained on an outdoor turfed field and an indoor multipurpose sport surface. However, for best practice, practitioners should seek to limit as many extraneous variables as possible and, as a result, should seek to standardize their testing practice, including surface type. In this way, future research should look to monitor indoor and outdoor environmental conditions, including but not limited to temperature, humidity, solar exposure, and surface condition (i.e., grass length/type, dew, and/or water pooling). We recognize this as a limitation of our study, where conditions such as grass length/type were not recorded. Moreover, researchers should not compare the results of run-based tests obtained on an outdoor turfed surface and an indoor multipurpose sport surface due to methodological issues regarding different mechanical properties (e.g., friction or energy return) and confounding factors (e.g., altered technique or loading patterns).

## 5. Conclusions

The results from our study suggest that the 10 m sprint, 20 m sprint, and MSFT have acceptable absolute (CV < 5%) and relative (ICC = *moderate-good*) reliability on an indoor multipurpose sport surface. Importantly, we found the 505-Agility test to reside outside the acceptable range of absolute reliability (left leg lead: 5.1%) and to have *poor* relative reliability with a right leg and left leg lead. Researchers should not compare results of run-based tests performed on outdoor turfed surfaces and indoor multipurpose surfaces to maintain best practice. However, if necessary, practitioners may compare the results of run-based tests performed on an outdoor turfed surface and an indoor multipurpose sport surface to gauge player performance or progression. Nevertheless, best practice dictates that practitioners should standardize their testing procedures.

## Figures and Tables

**Table 1:** Test–retest reliability of run-based tests performed on an indoor multipurpose sports surface.

Test	n	CV (%)	Acceptable CV	ICC (95% CI)	*ICC Classification*
10 m sprint	15	1.9	Yes	0.76 (0.43 to 0.91)	*Good*
20 m sprint	15	1.4	Yes	0.86 (0.63 to 0.95)	*Good*
505-Agility Test right	15	4.6	Yes	0.19 (−0.34 to 0.63)	*Poor*
505-Agility Test left	15	5.1	No	0.21 (−0.32 to 0.64)	*Poor*
Multistage fitness test (mL·kg^−1^·min^−1^)	15	4.1	Yes	0.63 (0.19 to 0.86)	*Moderate*

Abbreviations: n = sample; CV = coefficient of variation; ICC = intraclass correlation coefficient; CI = confidence interval. An acceptable CV of 5.0% was utilized for the 10 m sprint test, 20 m sprint test, and 505-Agility Test based on the recommendation of Currell et al. [25]. An acceptable CV of 10.0% was utilized for the multistage fitness test based on the recommendation of Currell et al. [25]. The ICC classifications were based on the ICC value independent of the 95% confidence interval [26]. The ICC classifications were defined based on the recommendations by Koo et al. [26] as follows: *poor:* ≤0.50, *moderate*: 0.50 to 0.74, *good*: 0.75 to 0.90, or *excellent*: ≥0.90.

**Table 2:** Assessment of differences in run-based tests occurring on an outdoor turfed field and an indoor multipurpose sport surface.

Test	n	Outdoor Turfed Surface	Indoor Multipurpose Sport Surface		Comparison of Run-Based Tests Conducted During Week 1 and Week 2			
		Week 1 Mean ± SD	Week 2 Mean ± SD	Week 3 Mean ± SD	*p*-Value	Hedges’ *g*	95% CI	Effect Size Magnitude
10 m sprint (s)	15	1.75 ± 0.05	1.77 ± 0.10	1.77 ± 0.11	0.56	0.15	−0.41 to 0.70	*Trivial*
20 m sprint (s)	15	3.05 ± 0.12	3.06 ± 0.16	3.07 ± 0.18	0.82	0.06	−0.49 to 0.61	*Trivial*
505-Agility right (s)	15	2.31 ± 0.20	2.20 ± 0.10	2.15 ± 0.17	0.05	−0.53	−1.12 to 0.06	*Trivial*
505-Agility left (s)	15	2.29 ± 0.18	2.21 ± 0.12	2.15 ± 0.19	0.13	−0.40	−0.97 to 0.17	*Trivial*
Multistage fitness test (mL·kg^−1^·min^−1^)	15	46.09 ± 3.82	47.4 ± 3.87	46.79 ± 5.45	0.33	0.25	−0.31 to 0.81	*Trivial*

Abbreviations: n = sample; SD = standard deviation. The *p*-value represents significance and was set *a priori* at *p* < 0.05. Hedges’ g is the effect size and represents the meaningfulness of the finding. Effect size magnitudes were determined as *trivial*: <0.20; *small*: 0.20–0.49; *medium*: 0.50–0.79; and *large*: ≥0.80 [28]. However, when the 95% overlapped 0 the effect size magnitude was determined as *trivial* [28].

## Data Availability

The data that support the findings of this study are available from the corresponding author upon reasonable request.
